# Regulation of gene expression through protein-metabolite interactions

**DOI:** 10.1038/s44324-024-00047-w

**Published:** 2025-03-04

**Authors:** Maximilian Hornisch, Ilaria Piazza

**Affiliations:** 1 Max Delbrück Center for Molecular Medicine in the Helmholtz Association, Robert-Rössle-Str. 10, Berlin, 13092 Germany; 2 SciLifeLab, Department of Microbiology, Tumor and Cell Biology, Karolinska Institutet, Solna, 171 65 Sweden

**Keywords:** Biochemistry, Chemical biology, Systems biology

## Abstract

Organisms have to adapt to changes in their environment. Cellular adaptation requires sensing, signalling and ultimately the activation of cellular programs. Metabolites are environmental signals that are sensed by proteins, such as metabolic enzymes, protein kinases and nuclear receptors. Recent studies have discovered novel metabolite sensors that function as gene regulatory proteins such as chromatin associated factors or RNA binding proteins. Due to their function in regulating gene expression, metabolite-induced allosteric control of these proteins facilitates a crosstalk between metabolism and gene expression. Here we discuss the direct control of gene regulatory processes by metabolites and recent progresses that expand our abilities to systematically characterize metabolite-protein interaction networks. Obtaining a profound map of such networks is of great interest for aiding metabolic disease treatment and drug target identification.

## Metabolites as regulators of gene expression

Organisms must rapidly adapt to environmental changes to survive. They maintain cellular homeostasis by adjusting cellular functions in response to external conditions. Metabolites, which are closely linked to the environment, provide a direct connection between external factors and cellular behavior. Across all domains of life, organisms have evolved various mechanisms to sense metabolites and adjust their cellular programs accordingly. For adaptation to occur, metabolite sensing must lead to changes in metabolic pathway activities. These pathways, comprising interconnected chemical reactions catalyzed by enzymes, are responsible for producing and consuming metabolites essential for cellular functions. The activity of these pathways can be regulated in two primary ways: by adjusting the abundance of metabolic enzymes through gene expression and by directly modifying the activity of these enzymes.

A well-established mechanism for regulating enzyme activity is through allosteric interactions, where a molecule distinct from the enzyme’s substrate induces a conformational change, thereby influencing the enzyme’s function^1^. This form of regulation, known as allostery, allows one ligand to control a protein’s function, which in turn affects the protein’s interaction with other biomolecules, such as DNA, RNA, or another protein^2^. In addition to allosteric regulation by transient interactions, metabolites can also influence proteins through post-translational modifications (PTMs), such as phosphorylation, methylation, O-GlcNAcylation or acetylation^3^. These covalent chemical tags, derived from metabolites, are linked to metabolite availability and can significantly impact gene expression^4^.

The mechanisms that link metabolic changes to gene expression, however, generally differ between bacteria and eukaryotes in terms of complexity and directness. In bacteria, metabolites often exert a direct influence on RNA and DNA-binding proteins that initiate gene expression changes. For example, bacterial transcription factors can often physically bind metabolites^5^, as can RNA riboswitches—molecular switches within RNA molecules that bind small metabolites and alter gene expression in response^6^. Eukaryotic organisms, on the other hand, typically rely on more complex and indirect mechanisms due to the packaging of their genomes in chromatin. In these cells, changes in intracellular metabolite concentrations often affect gene expression through intermediary steps, such as the regulation of epigenetic marks—covalent modifications of histone proteins and nucleic acids^4,7^. These marks serve as intermediate messengers that translate metabolic states into changes in gene expression. Nevertheless, eukaryotic cells also possess metabolite-sensing transcription factors, such as those in the nuclear receptor family, which can respond immediately to metabolic signals^8^.

This level of regulation of gene expression by metabolites, that involves transient interactions with gene regulatory proteins, represents one of the most immediate and specific mechanisms for linking metabolism to gene expression. These interactions are reversible and dynamic, allowing cells to quickly adapt to fluctuating metabolic conditions. In this review, we will explore the specific mechanisms of how transcriptional and translational processes are allosterically regulated by small molecule metabolites, and discuss methods available for discovering novel regulatory pathways through the study of metabolite-protein interactions.

## Metabolic regulation of the human nucleus: linking metabolites to chromatin function

In human cells the genome is sequestered within the nucleus, an organelle that physically separates the DNA tightly packed in the form of chromatin. This compartmentalization raises the critical question of how are metabolite levels regulated within the nucleus? One possibility is that metabolites can freely diffuse from the cytoplasm into the nucleus through nuclear pores, maintaining a concentration equilibrium between these compartments^9^. However, emerging evidence suggests that the nucleus may function as a metabolically distinct compartment^10,11^. Some metabolic enzymes are known to localize within the nucleus, where they directly influence nuclear processes^12–15^ playing a role in mammalian zygotic genome activation^16^ or the regulation of stem cell pluripotency^17^.

For instance, the enzyme ATP-citrate lyase, which converts acetate and Coenzyme A into Acetyl-CoA, and TCA cycle-related enzymes provide substrates for crucial nuclear reactions, including histone acetylation^18,19^ and DNA demethylation^20^. Moreover, metabolic enzymes can bind to chromatin at specific loci, as in the case of the one-carbon metabolic enzyme C1-tetrahydrofolate synthase MTHFD1^21^ and of the adenosylhomocysteinase AHCY^22^. When bound to specific genomic regions, these enzymes can locally facilitate metabolic processes that drive epigenetic reactions. For example, the acyl-CoA synthetase ACSS2 locally produces Acetyl-CoA for H3 acetylation, promoting the expression of lysosomal and autophagy genes^23^.

**Fig. 1:**
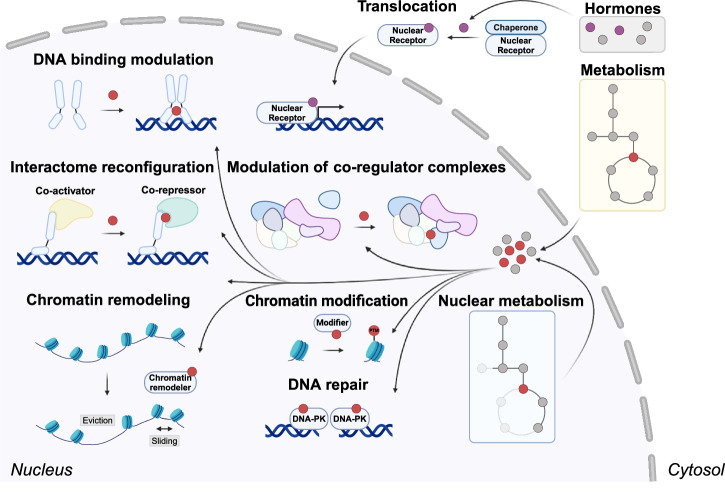
Mechanisms of metabolic regulation of chromatin function in the nucleus. Small molecule metabolites derived from exogenous sources or cellular chemical reactions regulate genome function, such as epigenetic modifications, chromatin remodeling and DNA repair. Metabolites can also act on the transcriptional machinery by affecting the translocation of nuclear receptors, by regulating DNA binding of transcription factors or by reconfiguring the interactome of transcription factors and co-regulator complexes. Key pathways include: (1) DNA-binding modulation—metabolites influence transcription factors, altering the DNA-binding capacity of the transcription factors IRF6, CLOCK1 and HIF3A for instance. (2) Interactome reconfiguration—the availability of metabolites modulates the interaction between transcription factors and co-regulators as well as between subunits of co-regulator complexes, affecting transcriptional outcomes. For instance, binding of thyroid hormones to the thyroid hormone receptor promotes binding of co-activators and suppresses co-repressor binding. (3) Chromatin remodeling—metabolite-regulated chromatin remodelers, which for example are regulated by inositol-phosphates, reposition nucleosomes, thereby affecting gene accessibility. (4) Chromatin modifications and DNA repair—metabolic enzymes localized in the nucleus contribute substrates for histone modifications and DNA repair processes. (5) Nuclear metabolism—certain metabolic enzymes are localized within the nucleus, providing essential intermediates for epigenetic modifications and other nuclear functions, such as the Acyl-CoA synthetase ACSS2 which locally produces Acetyl-CoA. (6) Translocation and exogenous molecules—the translocation of metabolites into the nucleus occurs via nuclear pores, while nuclear receptors respond to exogenous molecules, such as hormones and vitamins, linking external signals with nuclear functions.

Once metabolites enter the nucleus, they influence gene expression in two key ways. First, they control epigenetic processes by regulating the deposition and removal of epigenetic marks on histones and nucleic acids^7,24^. These modifications, orchestrated by epigenetic writers, readers, and erasers, play a pivotal role in governing genome functions such as replication, recombination, and transcription^25^ (Fig. 1). Second, metabolites can physically bind chromatin-associated proteins, acting as alternative substrates, products, or regulators. For instance, inositol phosphate metabolites bind chromatin remodelers like the SWI/SNF complex, modulating their activity^26^. Inositol phosphate metabolites also bind DNA repair enzymes to stimulate double-strand break repair^27,28^. ATP and lactate interact with proteins such as the barrier-to-autointegration factor (BAF)^29^ and the anaphase-promoting complex (APC)^30^, influencing DNA binding and mitotic progression (Fig. 1). These direct, transient interactions enable rapid and dynamic regulation of chromatin structure and function in response to metabolic changes.

### Regulation of transcription by metabolites

Transcription factors are the class of proteins with the most direct influence on gene expression. The regulation of transcription factors by metabolites establish a crucial link between metabolism and gene regulation. In human cells only a limited number of transcription factors are known to be metabolite-regulated, including those from the nuclear receptor superfamily, the basic helix-loop-helix PER-ARNT-SIM (bHLH-PAS) family and the sterol regulatory element binding protein (SREBP) family (Table 1). The nuclear receptor superfamily, with 48 members, is the most common class of ligand-regulated transcription factors. These proteins typically share a domain structure comprising an unstructured N-terminal domain, a DNA-binding zinc finger domain, a hinge region, and a ligand-binding domain^31^. Nuclear receptors bind to lipophilic molecules like steroid hormones, vitamin D, or fatty acids. Ligand binding can lead to various functional outcomes, including translocation to the nucleus and interaction with co-regulators, which modulate transcription (Fig. 1).

**Table 1:** Metabolite-regulate transcription factors

	Protein family	Transcription factor	Mechanism	Ligand	Function
Ligand-controlled transcription factors	Nuclear receptor protein family (*n* = 48)	PARa, RXRa, AR, …	Ligands bind to ligand-binding domain which triggers nuclear translocation or reconfiguration of interactome	Hormones, Fatty acids, Vitamins	Regulation of energy metabolism
	bHLH-PAS family (*n* = 16)	CLOCK1	NAD(H)/NADP(H) binding affects DNA binding	NAD(H)/NADP(H)	Regulation of circadian rhythm
			Heme binding modulates heterodimerization with BMAL1	Heme	
		NPAS2	NAD(H)/NADP(H) binding affects DNA binding	NAD(H)/NADP(H)	Regulation of circadian rhythm
			CO-Heme binding modulates heterodimerization with BMAL1	Heme, CO	
		HIF3a	OEA binds to HIF3a and promotes heterodimerization with ARNT	Oleoylethanolamide (OEA)	Unknown
	IRF family (*n* = 9)	IRF6	Glucose binding enhances homodimerization	Glucose	Regulation of keratinocyte differentiation
	C2H2 transcription factor family (*n* = 614)	MTF-1	Zinc stabilizes zinc finger domains, exact mechanism unknown	Zinc	Regulation of metal homeostasis
	SREBP family (*n* = 2)	SREBP1/2	SREBP is released from the ER membrane in and sterol-dependent manner and translocates in the nucleus.	Sterol	Regulation of lipid metabolism
	Mondo family (*n* = 2)	ChREBP	Glucose promotes translocation to nucleus, exact mechanism unknown	Carbohydrates (Glucose-6-phosphate)	Regulation of energy metabolism
		MLX	Glucose promotes translocation to nucleus, exact mechanism unknown	Carbohydrates (Glucose-6-phosphate)	Regulation of energy metabolism
Stability	bZIP family (*n* = 55)	NRF2	Itaconate increases stability of NRF2 by inhibiting KEAP1 via alkylation	Itaconate	Activation of anti-inflammatory program
	bHLH-PAS family (*n* = 16)	HIF1a	Oxygen affects HIF1a stability via hydroxylation	Oxygen	Regulation of cellular response to hypoxia
Co-regulators	D-Isomer specific 2-hyrdroxyacid dehydrogenase family (*n* = 4)	CTBP1	NAD(H) binding modulates interactions with transcription factors and acetyltransferases	NAD(H)	Regulation of acetyltransferases
	Glyeraldehyde-3-phosphate dehydrogenase family (*n* = 2)	GAPDH	NAD(H) binding modulates interaction with OCA-S complex	NAD(H)	Regulation of histone gene expression
	TRIM/RBCC family (*n* = 65)	TRIM28	Lactate interacts with coiled-coiled domain	Lactate	Unknown
	Histone deacetylase family (*n* = 11)	HDAC3	Inositol tetraphosphate stabilizes interaction with transcriptional repressors	Inositol tetraphosphate	Unknown

For example, steroid hormone receptors are stabilized by binding to hormones such as glucocorticoids, androgen or progesterone, which release the receptors from cytosolic chaperones, enabling their translocation to the nucleus^32^. In the nucleus, these receptors bind to hormone response elements on target genes, profoundly influencing chromatin structure and transcription to regulate biological functions such as reproduction and metabolism^8,33^. In contrast, thyroid hormone receptors bind to DNA in their ligand-unbound state, and ligand binding induces a switch from recruiting transcriptional repressors to activators^34^, affecting energy metabolism and behavioral phenotypes such as exploration^35^.

Beyond nuclear receptors, other transcription factors in humans are regulated by metabolites. For instance, members of the bHLH-PAS transcription factor family, such as CLOCK1, NPAS2 and HIF3A have PAS domains that can sense various small molecules like heme^36^, carbon monoxide^37^ or lipids^38,39^, influencing dimerization and DNA binding. The CLOCK1-BMAL1 and NPAS2-BMAL1 transcription factor complexes bind NAD(H)/NADP(H) in their DNA-binding domains, with the redox state modulating their transcriptional activity^40^. Additionally, the human interferon-regulatory factor 6 (IRF6), binds glucose via its DNA-binding domain, with glucose influencing IRF6’s ability to dimerize and bind DNA, thereby activating an epidermal differentiation program in keratinocytes^41^. Similarly, the C2H2 zinc finger MTF-1 senses metal ions with its zinc finger domain, regulating metal homeostasis genes^42,43^.

**Fig. 2:**
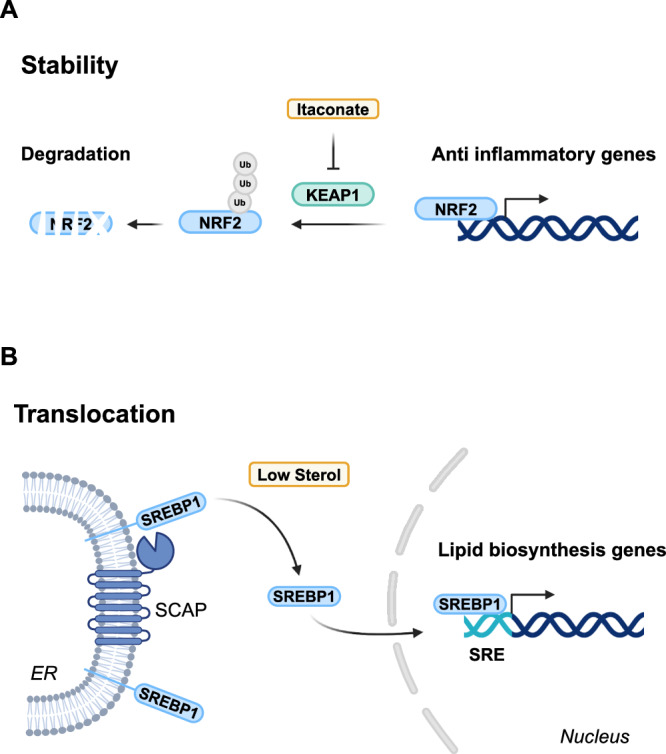
Regulation of transcription factors by metabolite sensors. **A** The metabolite itaconate inhibits KEAP1 preventing degradation of NRF2 which activates genes responsible for an anti-inflammatory response. KEAP1 Kelch-like ECH-associated protein 1, NRF2 nuclear factor erythroid 2-related factor 2, Ub ubiquitin. **B** SCAP proteolytically releases a transcriptionally active fragment of SREBP1 under low sterol conditions which subsequently translocates to the nucleus regulating the expression of lipid biosynthesis genes. ER endoplasmic reticulum, SREBP1 sterol regulatory-element binding protein, SCAP SREBP cleavage-activating protein, SRE sterol response element.

Transcription factors that sense metabolites through direct binding are intrinsically regulated by small molecules. Others, however, are more indirectly regulated by physically interacting with metabolite-sensing proteins, such as post-transcriptional modifiers^44^, proteases^45^ or E3 ligase complexes^46^. For instance, HIF1a, a bHLH-PAS transcription factor is hydroxylated in an oxygen-dependent manner. Under normoxic conditions, hydroxylation directs HIF1A for degradation, whereas in hypoxia reduced hydroxylation allows HIF1A to accumulate, translocate to the nucleus and activate hypoxia response genes^47^. Similarly, the metabolite itaconate regulates the transcription factor NRF2 by inhibiting its degradation, leading to the activation of anti-inflammatory programs in macrophages^46^ (Fig. 2A). Transcription factors like SREBP1/2 are regulated by proteolytic release and translocation from the endoplasmic reticulum membrane under low sterol conditions (Fig. 2B), while translocation from the cytosol to the nucleus of the Mondo transcription factors (ChREBP and MondoA) is regulated by carbohydrates, modulating lipid biosynthesis^45,48^ and energy metabolism gene expression^49,50^ respectively.

Once in the nucleus and bound to DNA, transcription factors can recruit co-regulators that either repress or activate transcription through chromatin remodeling or chemical modification or by directly engaging the transcriptional machinery. These co-regulators typically assemble in complexes with transcription factors through protein-protein interactions which can be modulated by metabolites^51^. For instance, CTBP1 represses transcription by inhibiting histone acetyltransferases, and this activity is regulated by NADH, linking cellular energy levels to transcriptional regulation^52,53^. Similarly, the metabolic enzyme GAPDH’s role in transcriptional activation is regulated by the NAD/NAD(H) ratio, which modulates its incorporation into the OCA-S complex, affecting the expression of histone genes during the cell cycle^54^. Additional examples include the co-repressor TRIM28, which is regulated by lactate (Tian et al.^55^), and the histone deacetylase HDAC3 whose interaction with co-repressors is stabilized by inositol phosphates^56^.

Once transcribed, RNA molecules undergo post-transcriptional processing steps, such as splicing, nuclear export, and translation. The question remains: Can metabolites also influence these processes?

### Crosstalk between RNA function and metabolism

**Fig. 3:**
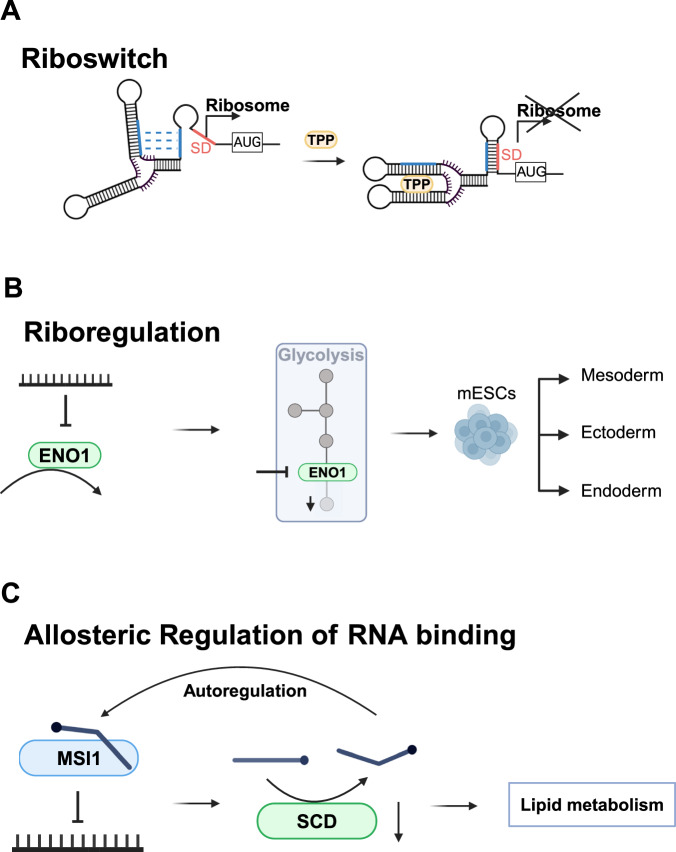
Regulation of RNA function by metabolites. Metabolites can affect translation by binding to riboswitches. **A** Binding of the metabolite thiamine pyrophosphate (TPP) to the thiM riboswitch induces a conformational change masking the Shine-Dalgarno sequence (SD) inhibiting ribosome binding and translation initiation. **B** RNA molecules can inhibit metabolic enzymes such as the glycolytic enzyme ENO1 which is relevant for mESC differentiation. **C** Metabolites can allosterically regulate RNA-binding proteins. Unsaturated lipids allosterically inhibit RNA binding of MSI1 which leads to the translational down-regulation of the lipid biosynthesis enzyme SCD and to changes in lipid metabolism. mESC mouse embryonic stem cells, MSI1 Musashi 1, SCD steaoryl-Coa desaturase 1.

RNA molecules play crucial roles in sensing and regulating metabolic processes within the cell. One way this occurs is through riboswitches, which are specific RNA structures that respond to metabolite binding to regulate transcription, translation, and alternative splicing. Typically located in the 5’ untranslated regions (UTRs) of mRNAs, riboswitches consist of a ligand-binding aptamer and juxtaposed RNA sequences that are important for translation initiation such as the Shine-Dalgarno sequence or translation termination sequences^57^. When a ligand binds a riboswitch, it induces an RNA conformational change that affects the accessibility of the adjacent regulatory RNA sequences, often serving as a feedback mechanism in metabolic pathways^58^. For instance, the *E.coli* thiM mRNA, which encodes a metabolic enzyme involved in thiamine phosphate biosynthesis, carries a riboswitch that binds the co-factor thiamine pyrophosphate (TPP). Upon TPP binding the thiM riboswitch changes conformation impeding ribosome binding, thus linking TPP levels to expression of thiamine phosphate biosynthesis genes^59^ (Fig. 3A). Although more than 55 riboswitch classes have been identified, only one has been found in eukaryotes, the TPP riboswitch class, and none in vertebrates^60^, raising the question: How do eukaryotes integrate metabolic signals into their post-transcriptional gene regulation programs?

### Metabolic enzymes binding RNA

One answer lies in the discovery of RNA-binding proteins (RBPs) that are regulated by metabolites. For example, the iron-regulatory protein 1 (IRP1), which binds RNA in response to iron levels, regulates iron homeostasis in eukaryotes. IRP1 binds to specific RNA structures in the UTRs of mRNAs encoding iron metabolism proteins, adjusting their expression in response to iron scarcity. Interestingly, IRP1 is also a cytoplasmic homolog of the mitochondrial enzyme aconitase, exemplifying a “moonlighting” function where a metabolic enzyme also serves as an RBP^61^. This dual function—as metabolite- and RNA-interactor—is not unique to IRP1; other metabolic enzymes also facilitate crosstalk between metabolism and RNA function^62–66^. By regulating the RNA-binding activity of these enzymes, metabolites can influence the stability or translation of target RNAs. For instance, in T-cell activation, a metabolic switch modulates the RNA-binding activity of GAPDH, controlling the translation of an immune cytokine^67^ and the autoregulation of thymidylate synthase expression^68^. Additionally, RNA molecules can regulate the activity of metabolic enzymes, a phenomenon known as “riboregulation”^63^. An example of this occurs in glycolysis, where RNA inhibits the catalytic activity of enolase, playing a significant role in cell differentiation^69^ (Fig. 3B).

Advances in RNA-binding protein capture techniques combined with proteomics have revealed that many more metabolic enzymes bind RNA across various species, both in cell culture models and tissues^70–74^. These proteomics-based studies provide evidence for RNA binding of more than 40% of all metabolic enzymes^75^. These findings raise important questions: How do these enzymes interact with RNA? Do they bind specific RNA sequences, or is their binding nonspecific? And what are the cellular functions of these interactions? Initial studies mapping RNA-binding regions in proteins have shown that metabolic enzymes tend to interact with RNA near their active sites, often through nucleotide-binding domains such as the Rossmann fold^76,77^. However, understanding the biochemical details of these interactions requires structural insights. Recently, the structure of the metabolic enzyme SHMT1 bound to RNA was resolved, confirming its interaction with RNA close to its active site^78^.

### Modulation of canonical RNA binding protein activity by metabolites

Canonical RNA-binding proteins, which typically have well-characterized RNA-binding domains, are increasingly recognized as potential metabolite sensors^79^. For example, Musashi 1 (MSI1), a well-known RBP, was found to have its RNA-binding activity allosterically inhibited by fatty acids, which in turn affects the translation of lipid biosynthesis genes^80^ (Fig. 3C). Similarly, other metabolites such as UDP-glucose and ATP have been shown to regulate the activity of canonical RBPs like HuR^81^, FUS^82,83^, CIRBP^84^, TDP-43^85^, and HNRNPA1^86^ with these interactions impacting processes such as epithelial-to-mesenchymal transition^81^ and the formation of phase condensates^84^.

In addition to these examples, the amino acid arginine has been found to interact with the RNA-binding protein RBM39, which controls the expression of metabolic genes in liver cancer cells, thereby promoting tumor formation^87^. Arginine also interacts with RNA-binding proteins involved in mRNA splicing, such as RNA helicases^88^, although the impact of this interaction on gene expression is not fully understood. In general, the interactions between RNA-binding proteins and metabolites can play a significant role in splicing regulation^89^. For example, glucose binds to the ATP-binding site of the RNA helicase DDX21, inhibiting its ability to form homodimers. This leads to the incorporation of DDX21 into splicing complexes involved in epidermal differentiation^90^, further emphasizing the emerging role of splicing as a target for metabolic regulation.

Interestingly, RNA-binding domains in proteins like HuR and CIRBP play a crucial role in metabolite interactions. The RRM domain in HuR binds UDP-glucose, preventing RNA interaction and altering gene expression^81^. Similarly, ATP binds to CIRBP’s RRM domain, competing with RNA in vitro^84^. However, it remains unclear how common it is for metabolites to bind directly to canonical RNA-binding domains or other regions in RBPs. Clarifying these structural details is crucial for understanding how RNA-binding domains and other RBPs regions integrate metabolic signals and influence post-transcriptional processes. This understanding also has significant implications for drug development. Several compounds have been designed to modulate RNA-binding protein interactions^91,92^. For instance, inhibitors targeting the RNA-binding protein HuR, such as the small molecule MS-444, prevent HuR from binding to its RNA targets, which has shown promise in reducing tumor growth in certain cancers^93^ and small molecule modulators of splicing, such as Risidiplam that has been approved for the treatment of spinal muscular atrophy^94^. These examples highlight the therapeutic potential of targeting RNA-binding proteins and their interactions with metabolites or RNA.

### Methods for characterizing metabolite-protein interactions

**Fig. 4:**
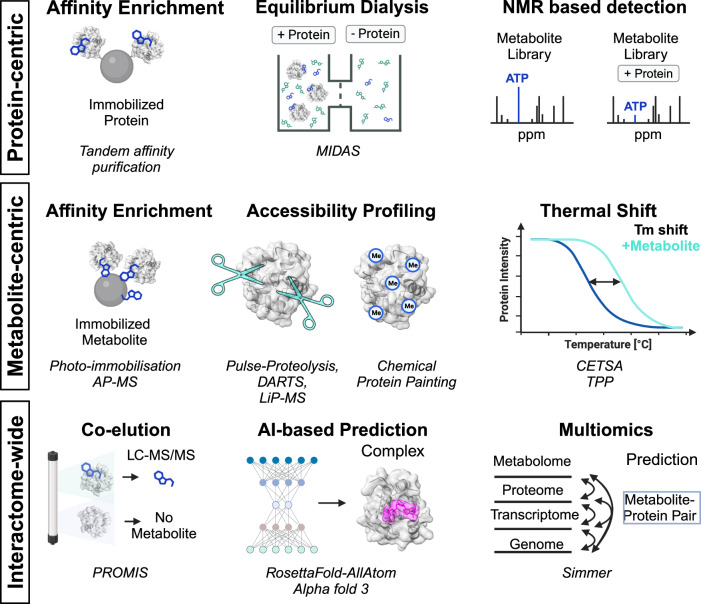
Methods for characterizing metabolite-protein interactions. Summary of metabolite-protein interaction profiling technique categorized in protein-centric, metabolite-centric and interactome-wide approaches. MIDAS mass spectrometry integrated with equilibrium dialysis for the discovery of allostery systematically, NMR nuclear magnetic resonance, AP-MS affinity purification-mass spectrometry, DARTS drug affinity responsive target stability, LiP-MS limited-proteolysis coupled to mass spectrometry, CETSA cellular thermal shift assay, TPP thermal proteome profiling, Tm melting temperature, PROMIS protein-metabolite interaction using size selection, LC-MS/MS liquid chromatography tandem mass spectrometry, AI artificial intelligence, SIMMER systematic identification of meaningful metabolic enzyme regulation.

There are well-established methods for systematically profiling protein-protein, nucleic acid-protein, and drug-protein interactions. However, profiling metabolite-protein interactions remains particularly challenging, due to key differences in their chemical properties. Metabolite-protein interactions often have low affinity, which complicates detection. Additionally, the chemical diversity of metabolites makes designing a universal profiling strategy difficult. Furthermore, their small size makes them hard to modify while retaining activity. Therefore, effective methods must capture low-affinity interactions, be agnostic to metabolite chemistry, and avoid the need for modification. Recently, several tools have been developed that meet these criteria (also reviewed here^95–99^). Here, we provide an update on the most recent developments (Fig. 4).

Pull-down strategies have been effective for characterizing protein-protein^100^ and drug-protein interactions^101,102^, but are less commonly used for interrogating metabolite-protein interactions. This is partly due to the difficulty of immobilizing small and chemically diverse metabolites. However, photo-immobilization using diazirine photochemistry has addressed some of these challenges^103,104^. Upon UV irradiation, diazirines convert into carbenes, which readily insert into C-H bonds and other chemical groups^105^, allowing metabolites to be immobilized without prior modification. This method has been used to profile the interactomes of natural products and co-factors in both human and E. coli proteomes, showing FAD binding of a subset of human RNA-binding proteins^106^. While this approach enhances metabolite-centric pull-downs, it still relies on metabolite immobilization. Alternatively, protein tagging allows for the enrichment of proteins via affinity purification, with bound metabolites identified through LC-MS/MS^107^. However, this approach still requires modification of the protein bait into a fusion protein with an affinity tag.

Modern chemical proteomics workflows now focus on individual metabolites and their interactomes within the entire proteome. These methods exploit the principle that metabolite binding alters the biophysical properties of proteins. One such change is the shift in protein melting temperature when a ligand binds. The cellular thermal shift assay (CETSA) was first introduced to measure ligand-induced shifts in protein thermal stability^108^. This approach has since been adapted for use with mass spectrometry-based proteomics for protein quantification in techniques like thermal proteome profiling (TPP)^109^ or thermal stability profiling^110^. TPP has been successfully applied to map the ATP-protein interactome^29^ and the lactate-protein interactome discovering its role in the regulation of the anaphase-promoting complex^30^. Moreover, TPP has identified metal-binding proteins in lysates^111,112^ and allowed the detection of changes in metabolite-protein interactions in living cells^113^. Ligand binding also affects protein structural states, which can be detected through proteolysis using broad-spectrum proteases. Ligand-bound proteins are more resistant to proteolysis, a principle exploited in techniques like drug affinity responsive target stability (DARTS)^114^ and limited proteolysis coupled to mass spectrometry (LiP-MS)^115^. Chemical footprinting techniques further assess conformational changes by detecting alterations in the chemical reactivity of solvent exposed amino acid side chains^116^.

These chemoproteomics approaches have been used in various studies to map metabolite-protein interactomes, such as central carbon metabolites in E. coli^117^ and glycolytic metabolites in mammalian cell lysates^55^ and for profiling the ATP interactome in yeast^118,119^. While TPP generally provides insights into a larger fraction of the human proteome, LiP-MS and chemical footprinting offer spatial resolution, pinpointing specific protein regions involved in metabolite binding. Since chemical proteomics methods provide insights into the interactome of a single metabolite, profiling multiple metabolites is time-consuming and costly. However, advances in high-throughput proteomics are making large-scale mapping of metabolite-protein interactions increasingly feasible^120–123^.

Mapping metabolite-protein interactions on a large scale is possible, though it often involves analyzing one recombinant protein at a time using mass spectrometry-based metabolomics^124^ or nuclear magnetic resonance^125,126^. One notable technique is MIDAS (Mass spectrometry integrated with equilibrium dialysis for the discovery of allostery systematically), which has been used to discover allosteric regulators of enzymes involved in human carbohydrate metabolism^127^. MIDAS works by using equilibrium dialysis: a library of 401 metabolites is added to two chambers separated by a dialysis membrane. One chamber contains the purified protein, which is too large to diffuse across the membrane. Metabolites can equilibrate freely between the chambers, but those that bind to the protein accumulate in the protein-containing chamber. This allows researchers to identify metabolite-protein interactions by comparing the metabolite concentrations between the two chambers using mass spectrometry^124,127^. While MIDAS is effective for screening large numbers of metabolites, it is limited to proteins that can be purified with high purity. Another method, PROMIS, does not require protein purification and instead uses size-exclusion chromatography followed by proteomics and metabolomics analyses to infer metabolite-protein interactions^128^.

In contrast to purely experimental approaches, structural bioinformatics and artificial intelligence (AI) offer new ways to predict metabolite-protein interactions. Advances such as RoseTTAFold ALL-Atom^129^ and AlphaFold 3^130^ provide frameworks for predicting interactions across proteomes. Although these AI-based methods have shown promise, they require further experimental validation.

All methods discussed here infer metabolite-protein interactions from biochemical and biophysical experiments or computational models. However, these approaches are often limited as they do not manipulate metabolite levels in vivo. The complexity of living cells or organisms makes it challenging to separate effects due to metabolite-protein interactions from downstream events. Instead, combining *-omics* technologies and mathematical modeling can help disentangle these effects^131^, as demonstrated in studies that predict the protein metabolite network from measurement of metabolite levels and transcription factor activity^132,133^.

## Future perspective

While significant progress has been made in understanding how metabolism influences gene expression, a comprehensive overview of the crosstalk between metabolism and the proteome in human biology remains elusive. Throughout this review, we have highlighted numerous examples demonstrating that such a link exists, explored the underlying mechanisms, and discussed how pervasively metabolites regulate multiple layers of gene expression—including transcription^8,40,41,46,48^, RNA splicing^89^, and translation^79,80^. In bacteria, much more is known about the metabolome-proteome network. For example, approximately 30% of the 300 transcription factors in *E. coli*^5^ are allosterically regulated by metabolites, whereas in humans, only around 3% of the 1,600 transcription factors have been shown to engage in similar interactions^134^. This difference reflects the fundamentally distinct regulatory landscapes of bacteria and humans. In prokaryotes, the absence of compartmentalization, chromatin packaging, and epigenetic mechanisms allows for a more direct and immediate integration of metabolic signals into gene regulation. In humans, mechanisms such as epigenetic modifications, though crucial for gene regulation, tend to be more indirect compared to the small molecule regulation of transcription factors in bacteria. However, despite these differences, the basic logic of metabolite-induced modulation of DNA binding and protein-protein interactions is conserved across species. This suggests that metabolite-sensing transcription factors may also be more widespread in humans than currently recognized, with many yet to be discovered.

In addition, recent work has highlighted the intricate link between metabolism and translation, revealing how metabolic pathways not only provide energy for protein synthesis but also regulate the selectivity and efficiency of the translational machinery. For example, metabolic states, such as nutrient availability, can directly influence mRNA translation through pathways like mTOR and AMPK, which modulate key initiation factors^79^. Furthermore, metabolites themselves can act as signaling molecules, altering translational control to meet the demands of cellular physiology.

Technological advances now enable systematic searches for these regulatory interactions using a combination of experimental and computational approaches. We speculate that mapping the human metabolite-protein interactome in greater detail will reveal novel pathways linking metabolism to gene expression via gene regulatory proteins, such as metabolite-controlled transcription factors, chromatin remodelers, splicing regulators, and RNA-binding proteins. These interactions will likely have allosteric effects on protein functions, influencing nucleic acid binding, enzymatic activity, protein translation or protein-protein interactions. Therefore, it is essential not only to profile metabolite-protein interactions but also to investigate their impact on broader biomolecular networks.

Taken together, understanding these interactions will provide new insights into the complex regulatory networks that integrate metabolism with gene expression, potentially uncovering novel therapeutic targets in diseases where metabolism and gene regulation are dysregulated.

## Data Availability

No datasets were generated or analyzed during the current study.

## List of Abbreviations

ACSS2: Acetyl-coenzyme A synthetase 2
AHCY: Adenosylhomocysteinase
AI: Artificial intelligence
AMPK: AMP-activated protein kinase
APC: Anaphase promoting complex
ATP: Adenosine triphosphate
BAF: Barrier-to-autointegration factor
bHLH-PAS: Basic helix-loop-helix PER-ARNT-SIM
BMAL1: Basic helix-loop-helix ARNT-like protein 1
bZIP: Basic leucine zipper
CETSA: Cellular thermal shift assay
ChREBP: Carbohydrate response element binding protein
CIRBP: Cold-inducible RNA-binding protein
CLOCK: Circadian locomotor output cycles kaput
CoA: Coenzyme A
CTBP1: C-terminal binding protein 1
DARTS: Drug affinity responsive target stability
DDX21: DEAD box protein 2
ENO1: Enolase 1
FAD: Flavin adenine dinucleotide
FUS: RNA-binding protein FUS
GAPDH: Glyceradehyde-3-phosphate dehydrogenase
HDAC3: Histone deacetylase 3
HNRNPA1: Heterogenous nuclear ribonucleoprotein A1
HIF1a: Hypoxia-inducible factor 1 alpha
HIF3a: Hypoxia-inducible factor 3 alpha
HuR: Hu-antigen R
IRF6: Interferon-regulatory factor 6
IRP1: Iron-regulatory protein 1
KEAP1: Kelch-like ECH-associated protein 1
LC-MS/MS: Liquid chromatography with tandem mass spectrometry
LiP-MS: Limited-proteolysis coupled to mass spectrometry
mESC: Mouse embryonic stem cells
MLX: Max-like protein X
MIDAS: Mass spectrometry integrated with equilibrium dialysis for the discovery of allostery systematically)
MSI1: Musashi 1
MTHFD1: C-1-tetrahydrofolate synthase
MTF-1: Metal regulatory transcription factor 1
mTOR: Mammalian target of rapamycin
NAD: Nicotinamide adenine dinucleotide
NADP: Nicotinamide adenine dinucleotide phosphate
NMR: Nuclear magnetic resonance
NPAS2: Neuronal PAS domain protein 2
NRF2: Nuclear factor erythroid 2-related factor 2
OCA-S: Oct-1 coactivator in S-phase
PROMIS: Protein-metabolite interaction using size selection
PTM: Post-translational modification
RBP: RNA-binding protein
RRM: RNA-recognition motif
SCAP: SREBP cleavage-activating protein
SCD: Steaoryl-Coa desaturase 1
SHMT1: Serine hydroxymethyltransferase 1
SIMMER: Systematic identification of meaningful metabolic enzyme regulation
SRE: Sterol response element
SREBP: Sterol regulatory element binding protein
SWI/SNF: Switch/sucrose non-fermentable
TCA: Tricarboxylic acid
TDP-43: TAR DNA-binding protein 43
TPP (Metabolite): Thiamin pyrophosphate
TPP (Method): Thermal proteome profiling
TRIM28: Transcription intermediary factor 1 beta
TRIM/RBCC: Emerging tripartite motif/N-terminal RING finger/B-box/coiled-coil
UTR: Untranslated region
